# Real-Time Elastography: A Web-Based Nomogram Improves the Preoperative Prediction of Central Lymph Node Metastasis in cN0 PTC

**DOI:** 10.3389/fonc.2021.755273

**Published:** 2022-01-13

**Authors:** Chunwang Huang, Wenxiao Yan, Shumei Zhang, Yanping Wu, Hantao Guo, Kunming Liang, Wuzheng Xia, Shuzhen Cong

**Affiliations:** ^1^ Department of Ultrasound, Guangdong Provincial People’s Hospital, Guangdong Academy of Medical Sciences, Guangzhou, China; ^2^ The Second School of Clinical Medicine, Southern Medical University, Guangzhou, China; ^3^ Department of Ultrasound, Guangzhou Eighth People’s Hospital, Guangzhou Medical University, Guangzhou, China; ^4^ Department of Pathology, Guangdong Provincial People’s Hospital, Guangdong Academy of Medical Sciences, Guangzhou, China; ^5^ Department of Organ Transplant, Guangdong Provincial People’s Hospital, Guangdong Academy of Medical Sciences, Guangzhou, China

**Keywords:** ultrasonography, papillary thyroid carcinoma, clinically node-negative, central lymph node metastasis (CLNM), real-time elastography (RTE), nomogram

## Abstract

**Background:**

Given the difficulty of accurately determining the central lymph node metastasis (CLNM) status of patients with clinically node-negative (cN0) papillary thyroid carcinoma (PTC) before surgery, this study aims to combine real-time elastography (RTE) and conventional ultrasound (US) features with clinical features. The information is combined to construct and verify the nomogram to foresee the risk of CLNM in patients with cN0 PTC and to develop a network-based nomogram.

**Methods:**

From January 2018 to February 2020, 1,157 consecutive cases of cN0 PTC after thyroidectomy and central compartment neck dissection were retrospectively analyzed. The patients were indiscriminately allocated (2:1) to a training cohort (771 patients) and validation cohort (386 patients). Multivariate logistic regression analysis of US characteristics and clinical information in the training cohort was performed to screen for CLNM risk predictors. RTE data were included to construct prediction model 1 but were excluded when constructing model 2. DeLong’s test was used to select a forecast model with better receiver operator characteristic curve performance to establish a web-based nomogram. The clinical applicability, discrimination, and calibration of the preferable prediction model were assessed.

**Results:**

Multivariate regression analysis showed that age, sex, tumor size, bilateral tumors, the number of tumor contacting surfaces, chronic lymphocytic thyroiditis, and RTE were risk predictors of CLNM in cN0 PTC patients, which constituted prediction model 1. Model 2 included the first six risk predictors. Comparison of the areas under the curves of the two models showed that model 1 had better prediction performance (training set 0.798 vs. 0.733, validation set 0.792 vs. 0.715, *p* < 0.001) and good discrimination and calibration. RTE contributed significantly to the performance of the prediction model. Decision curve analysis showed that patients could obtain good net benefits with the application of model 1.

**Conclusion:**

A noninvasive web-based nomogram combining US characteristics and clinical risk factors was developed in the research. RTE could improve the prediction accuracy of the model. The dynamic nomogram has good performance in predicting the probability of CLNM in cN0 PTC patients.

## Introduction

Although papillary thyroid carcinoma (PTC) has an indolent clinical process, it is still prone to cervical lymph node (LN) metastasis, especially central LN metastasis (CLNM) in the beginning. CLNM is closely related to distant metastasis, recurrence, and a decreased survival rate (1–3). CLNM usually requires therapeutic central compartment neck dissection (CCND). However, whether prophylactic CCND should be enforced in clinically node-negative (cN0) patients remains controversial (4–7). Study has shown that 45% of cN0 PTC patients had CLNM after prophylactic CCND (8). Due to the benefits of prophylactic CCND for recurrence and survival and the selection of appropriate management strategies after surgery, some clinicians tend to choose prophylactic CCND (9–11). However, the weight of the potential dangers of prophylactic CCND, such as recurrent laryngeal nerve injury, permanent hypoparathyroidism, and other postoperative complications (4–7), cannot be simply overlooked. Therefore, a full understanding of the CLNM status of cN0 PTC patients before surgery is valuable for the selection of surgical resection methods and dissected range of LNs, thus contributing to the improvement in treatment strategies.

American Thyroid Association (ATA) Guidelines follow the principle of “less is more”. The guidelines, for example, recommend reducing the scope of surgery, fine-needle aspiration biopsy, radioactive iodine (RAI) for treatment or diagnosis, and follow-up examinations (12). In addition, immunotherapy emphasizes the importance of local LNs to survival because preservation of normal LNs is meaningful for subsequent treatment (13). Therefore, an accurate preoperative assessment of the CLNM risk is important for determining whether CCND should be implemented. However, diagnosing preoperative CLNM status in cN0 PTC is challenging and does not coincide with clinical needs. Therefore, an accurate, convenient, and noninvasive method to directly assess preoperative CLNM status is urgently needed (14).

Ultrasound (US) is the preferred imaging method for CLNM assessment and has the advantages of noninvasiveness, repeatability, and no radiation exposure. The sensitivity of US diagnosis of CLNM is not ideal because of interference from obesity (15) and adjacent organs (16–19). However, US characteristics are related to the invasiveness capability and adverse outcomes of PTC, and the results can guide the surgical strategy (5). Previous studies (20, 21) showed that the preoperative US characteristics of tumors can be used as high-risk predictors of CLNM in PTC. Therefore, a prediction model for the CLNM risk in cN0 PTC patients constructed based on preoperative US characteristics would have important guiding significance for selecting the surgical resection method, surgical scope, and management strategy.

As a common display tool for prediction models, nomograms increase the readability of prediction results and facilitate patient assessments by medical practitioners, which improves healthcare efficiency (22–25). Since a nomogram provides only approximate results and calculations are still required, the current trend is to use prediction models in the form of a mobile phone or tablet computer application or a web-based calculator (26). Some studies used clinical data and pathological features to construct nomograms to forecast CLNM in PTC, but most of the predictive factors were achieved after surgery (23, 25). Moreover, the sample sizes were comparatively small, and the prediction models were not validated. To date, no study has constructed a CLNM nomogram based on US characteristics for cN0 PTC patients and a web-based CLNM risk calculator.

The study was to create and validate a prediction model combining clinical data and preoperative US characteristics for CLNM in cN0 PTC patients and to generate a web-based nomogram to help clinicians adopt targeted and less damaging management strategies based on the risk of CLNM.

## Materials and Methods

### Study Population

**Figure 1:**
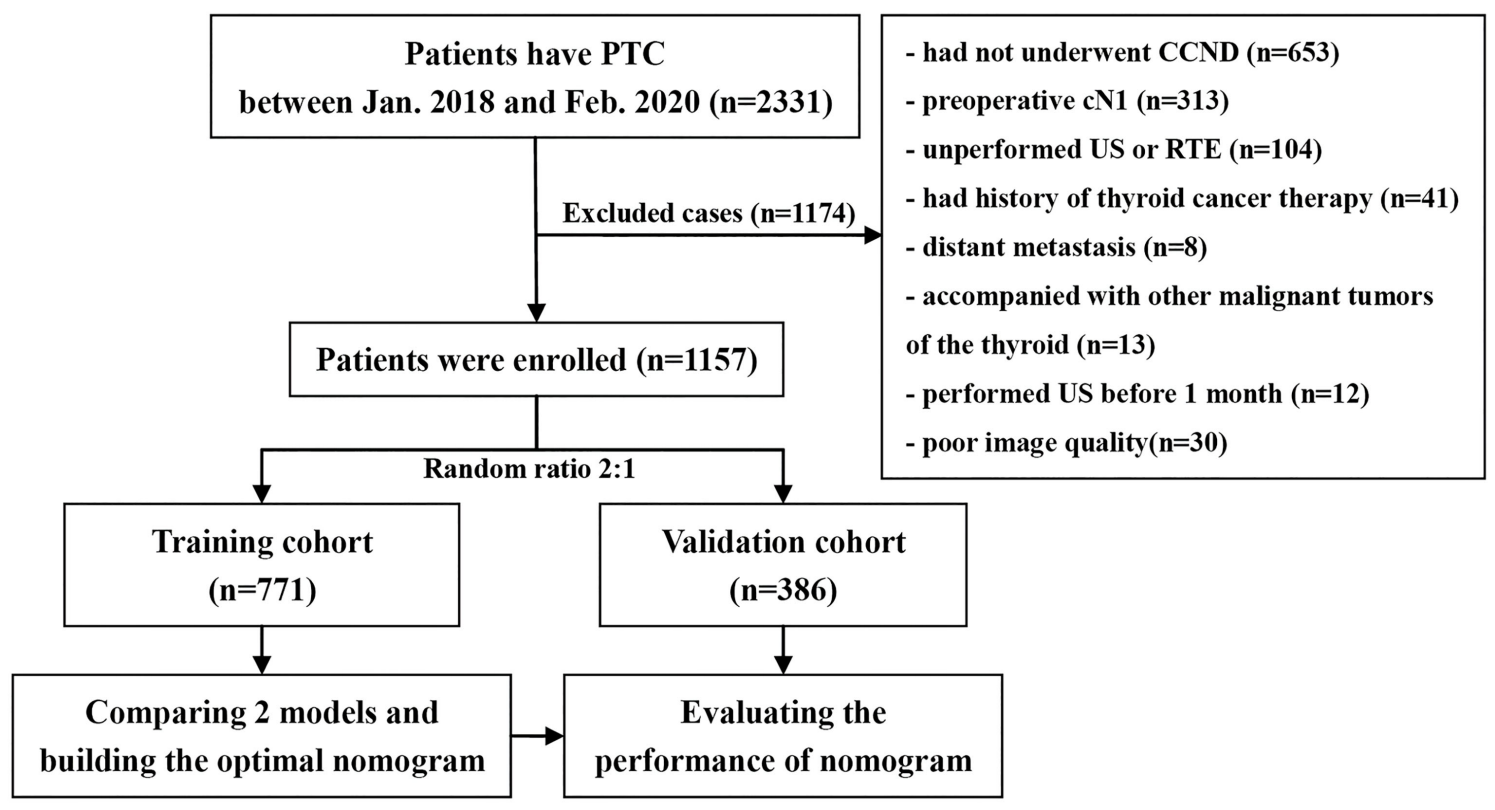
Flowchart of enrolled cN0 papillary thyroid carcinoma patients.

We retrospectively analyzed the databank of our medical institution from January 2018 to February 2020 and identified 1,157 consecutive cases of cN0 PTC with CCND for inclusion in the study. **Figure 1** shows the patient recruitment process. This study had a primary cohort and a validation dataset according to the results of random allocation (2:1); 771 patients (584 females and 187 males, mean age: 43.06 ± 9.50 years) were included in the training dataset, and 386 patients (285 females and 101 males, mean age: 41.43 ± 9.56 years) were included in the validation dataset. Clinical baseline data such as sex and age were collected from the database.

There were some selection criteria: (1) thyroid surgery was performed for the first time and PTC and the status of LNs were confirmed by histopathology; (2) cN0 is lymph node without metastasis diagnosed by preoperative imaging and CCND was performed; (3) thyroid US was accomplished in our department within 1 month before surgery and real-time elastography (RTE) was also conducted; and (4) complete medical data were accessible.

There were some exception criteria: (1) previous thyroid ablation and other related treatments or a history of surgery; (2) complicated with other thyroid malignancies; (3) distant metastasis or malignant tumors of other organs; (4) PTC or LN metastases that could not be clearly diagnosed by pathology; (5) tumors that could not be assessed by US; (6) the diagnosis, size and location of the tumor or largest tumor on US were not consistent with that on pathology; and (7) incomplete US information or poor image quality.

### US Images Evaluation and Definition

All ultrasound examinations were performed by color Doppler diagnostic tools (Hitachi, Ltd., Tokyo, Japan), with high frequency of 6–13 MHz linear array probe, and preoperative US data were stored for follow-up analysis.

Two experienced radiologists who have involved in thyroid imaging for 11 and 16 years individually retrospectively analyzed the US images. If a dispute arose between them, a third radiologist who has involved in thyroid imaging for 20 years participated in the discussion and made the final decision. These three radiologists only analyzed the US images and were unaware of other patient information.

The radiologists observed and recorded the sonographic characteristics of the tumor on US; when more than one suspicious thyroid malignancies were found, the relevant data of the largest suspicious tumor would be analyzed and recorded. US features including: (I) tumor size; (II) multifocality; (III) tumor position; (IV) bilateral tumor: 0 represents unilateral tumor/tumors, while 1 suggests bilateral multifocality; (V) echogenicity; (VI) calcification patterns: mode 0 represents no calcification, mode 1 represents microcalcification, and mode 2 represents coarse calcification. When coarse calcification and microcalcification coexisted in the lesion, it was classified as mode 1; (VII) number of contact surface (NCS), including anterior capsule, posterior capsule, upper pole, lower pole, medial, and lateral, was evaluated based on US images. Capsule contact was defined as over 25% of the tumor surface adjacent to or in contact with the thyroid capsule on US images or when the continuity of thyroid capsule echo around the tumor was interrupted. 0 means that the tumor is not associated with the capsule. 1 indicates that the tumor is associated with one side of the capsule, while 2 represents that the tumor contacted ≥ two sides of the capsule; (VIII) composition; (IX) shape; (IX) margin; (X) taller-than-wide; (XI) chronic lymphocytic thyroiditis (CLT); (XII) internal color Doppler vascularity: the evaluation criteria were based on Adler grade classification (27), which represents semiquantitative classification; and (XIII) cervical LN status: suspicious US features included increased cortical echo, cystic degeneration, microcalcification, and irregular or rich blood flow in LNs.

Routine US examination was followed by RTE, performed by the same radiologist. The same US machine and probe were used for elastography of thyroid nodules detected by conventional US. The elasticity score (ES) for each nodule would be assigned different points (0–4-point scale) according to the standards of Asteria et al. (28). The values of RTE were as follows: 0 for tumor ES 0–2; 1 for ES 3; and 2 for ES 4.

### Statistical Analysis

All tests were two sided with a statistical standard of 0.05. We used the Wilcoxon-Mann-Whitney *U* test or *t*-test to compare continuous variables and the Fisher’s exact test or Pearson’s *χ* ^2^ test for categorical variables. We used multivariable logistic regression analysis to screen US features and clinical data, determine risk predictors and regression coefficients, and constructed prediction models in the training dataset. We used the area under the receiver operating characteristic (ROC) curve (AUC) to measure the discriminative performance of the models in two datasets. DeLong’s test was used to compare models’ performance according to the AUC, the optimal model was adopted, and the nomogram was plotted. In addition, the calibration curve was drawn to weigh the calibration of the model for visualization in two cohorts. The probability of CLNM in each cN0 PTC patients (defined as nomo-risk in this study) was calculated on the basis of the nomogram calculation. The ideal cutoff point was decided by maximizing the Youden index. The AUC, specificity, sensitivity, likelihood ratios, and predictive values were calculated to weigh the performance of the optimal cutoff point of the nomo-risk. Decision curve analysis (DCA) was applied to quantify the net benefits of various threshold possibilities in the validation dataset and to evaluate the feasibility of using the optimal model in clinical practice (29). The “DynNom” and “shiny” packages of the R language were used to construct a web-based dynamic nomogram.

RStudio software (version 1.3.1073, RStudio Inc., Boston, MA, USA) and SPSS software (version 25.0, IBM Corp., Armonk, NY, USA) were used for statistical analysis.

## Results

### Clinical Baseline Data and US Characteristics

**Table 1** shows the clinical baseline data and US characteristics of the patients in the training dataset and the validation dataset. No significant difference in the prevalence of CLNM was found between the two datasets (*p* = 0.349). In addition, no significant difference in sex or age was identified among the CLNM-positive set and the CLNM-negative set between the two datasets, which confirmed the applicability of the two datasets used.

**Table 1:** Clinical and US features of cN0 PTC in the development and validation cohorts.

Characteristic	Development cohort (*n* = 771)			Validation cohort (*n* = 386)		
	CLNM(−) (*n* = 401)	CLNM(+) (*n* = 370)	*p*-value	CLNM(−) (*n* = 212)	CLNM(+) (*n* = 174)	*p*-value
Age (mean ± SD, range, years)	44.95 ± 9.49 (18–73)	41.01 ± 9.47 (14–73)	<0.001	43.40 ± 9.51 (19–70)	39.03 ± 9.45 (23–68)	<0.001
Sex (*n*, %)						
Female	327 (81.5)	257 (69.5)	<0.001	165 (77.8)	120 (69.0)	0.049
Male	74 (18.5)	113 (30.5)		47 (22.2)	54 (31.0)	
Tumor size (cm)	1.01 ± 0.50 (0.3–6.4)	1.37 ± 0.52 (0.3–7)	<0.001	0.99 ± 0.49 (0.3–6.3)	1.28 ± 0.50 (0.3–6.9)	<0.001
Tumor position (*n*, %)						
Left lobe	172 (42.9)	146 (39.4)	0.039	94 (44.3)	71 (40.8)	0.025
Right lobe	218 (54.4)	200 (54.1)		116 (54.7)	93 (53.4)	
Isthmus and conical lobe	11 (2.7)	24 (6.5)		2 (1.0)	10 (5.8)	
Multifocality (*n*, %)						
No	328 (81.8)	268 (72.4)	0.002	183 (86.3)	121 (69.5)	<0.001
Yes	73 (18.2)	102 (27.6)		29 (13.7)	53 (30.5)	
Bilateral tumors (*n*, %)						
No	358 (89.3)	286 (77.3)	<0.001	191 (90.1)	134 (77.0)	<0.001
Yes	43 (10.7)	84 (22.7)		21 (9.9)	40 (23.0)	
Very hypoechoic/hypoechoic (*n*, %)						
No	47 (11.7)	44 (11.9)	0.941	30 (14.2)	26 (14.9)	0.826
Yes	354 (88.3)	326 (88.1)		182 (85.8)	148 (85.1)	
Calcification (*n*, %)						
0	128 (31.9)	62 (16.8)	<0.001	68 (32.1)	31 (17.8)	0.003
1	250 (62.3)	292 (78.9)		133 (62.7)	137 (78.7)	
2	23 (5.8)	16 (4.3)		11 (5.2)	6 (3.5)	
NCS (*n*, %)						
0	208 (51.9)	124 (33.5)	<0.001	126 (59.4)	61 (35.1)	<0.001
1	161 (40.1)	159 (43.0)		69 (32.6)	74 (42.5)	
≥2	32 (8.0)	87 (23.5)		17 (8.0)	39 (22.4)	
Composition (*n*, %)						
Cystic	2 (0.5)	2 (0.5)	0.936	2 (0.9)	1 (0.6)	0.681
Solid	399 (99.5)	368 (99.5)		210 (99.1)	173 (99.4)	
Shape (*n*, %)						
Regular	14 (3.5)	13 (3.5)	0.987	12 (5.7)	5 (2.9)	0.184
Irregular	387 (96.5)	357 (96.5)		200 (94.3)	169 (97.1)	
Taller than wide (*n*, %)						
≤1	110 (27.4)	144 (38.9)	<0.001	57 (26.9)	62 (35.6)	0.064
>1	291 (72.6)	226 (61.1)		155 (73.1)	112 (64.4)	
Margin (*n*, %)						
Well-defined	19 (4.7)	15 (4.1)	0.644	8 (3.8)	9 (5.2)	0.505
Ill-defined	382 (95.3)	355 (95.9)		204 (96.2)	165 (94.8)	
CLT (*n*, %)						
Absence	253 (63.1)	271 (73.2)	0.003	140 (66.0)	112 (64.4)	0.732
Presence	148 (36.9)	99 (26.8)		72 (34.0)	62 (35.6)	
Internal vascularity (*n*, %)						
0	58 (14.5)	27 (7.3)	<0.001	35 (16.5)	13 (7.5)	<0.001
1	186 (46.4)	139 (37.6)		100 (47.2)	69 (39.7)	
2	74 (18.4)	90 (24.3)		35 (16.5)	54 (31.0)	
3	83 (20.7)	114 (30.8)		42 (19.8)	38 (21.8)	
RTE (*n*, %)						
0	57 (14.2)	14 (3.8)	<0.001	27 (12.7)	6 (3.4)	<0.001
1	316 (78.8)	231 (62.4)		172 (81.1)	103 (59.2)	
2	28 (7.0)	125 (33.8)		13 (6.2)	65 (37.4)	

NCS, number of contact surface; CLT, chronic lymphocytic thyroiditis; RTE, real-time elastography.

### Development and Comparison of the Prediction Models

Multivariate regression analysis demonstrated that sex, age, tumor size, multifocality, the NCS, CLT, and RTE were risk predictors, which constituted prediction model 1. Model 2 was composed of the first six risk predictors. **Table 2** shows the regression coefficients and odds ratios (ORs) of prediction models 1 and 2. In the two models, age and CLT were negatively correlated with CLNM, that is, a younger age corresponded to a greater likelihood of developing CLNM, while CLT corresponded to a reduced risk of CLNM development.

**Table 2:** Risk factors and regression coefficient of prediction models.

Intercept and variable	Model 1			Model 2		
	Coefficient	OR (95% CI)	*p*-value	Coefficient	OR (95% CI)	*p*-value
Intercept	−0.808	0.446 (0.175, 1.093)	0.083	0.224	1.251 (0.628, 2.496)	0.524
Sex						
Female		1 (reference)			1 (reference)	
Male	0.621	1.861 (1.264, 2.752)	0.002	0.665	1.944 (1.354, 2.802)	<0.001
Age	−0.034	0.967 (0.953, 0.981)	<0.001	−0.033	0.968 (0.955, 0.981)	<0.001
Tumor size	0.504	1.655 (1.258, 2.21)	<0.001	0.5	1.648 (1.261, 2.183)	<0.001
Bilateral tumors						
No		1 (reference)			1 (reference)	
Yes	0.859	2.362 (1.508, 3.738)	<0.001	0.786	2.194 (1.435, 3.388)	<0.001
NCS						
0		1 (reference)			1 (reference)	
1	0.575	1.777 (1.244, 2.548)	0.002	0.504	1.655 (1.189, 2.31)	0.003
2	1.148	3.153 (1.815, 5.556)	<0.001	1.152	3.164 (1.867, 5.438)	<0.001
CLT						
Absence		1 (reference)			1 (reference)	
Presence	−0.484	0.617 (0.43, 0.879)	0.008	−0.466	0.628 (0.449, 0.873)	0.006
RTE				NA	NA	NA
0		1 (reference)				
1	0.743	2.101 (1.119, 4.199)	0.027			
2	2.715	15.111 (7.263, 33.366)	<0.001			

OR, odds ratio; NCS, number of contact surface; CLT, chronic lymphocytic thyroiditis; RTE, real-time elastography; NA, not applicable.

**Figure 2:**
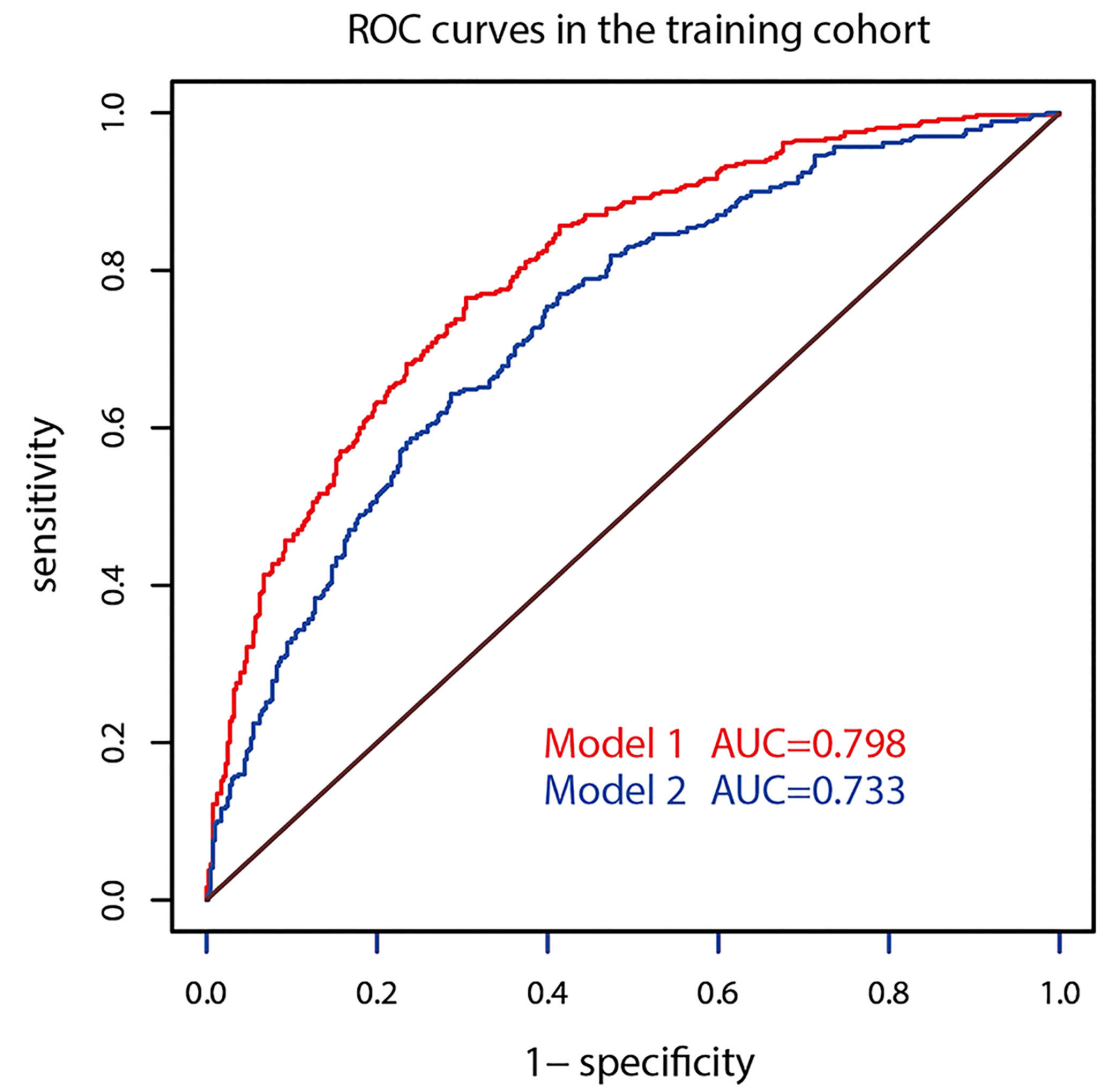
ROC curves of the two models in the training dataset.

**Figure 3:**
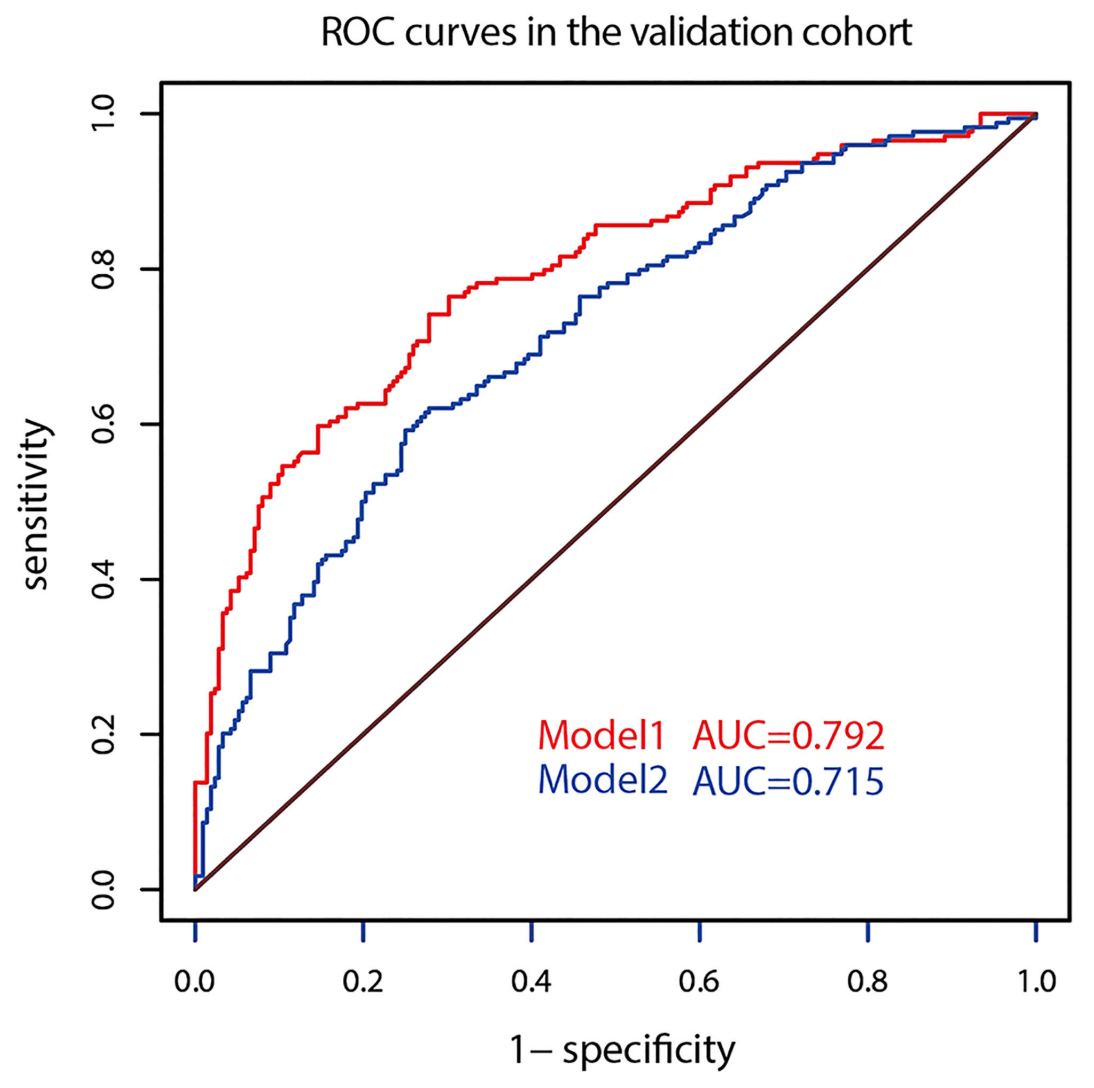
ROC curves of the two models in the validation dataset.

**Figure 4:**
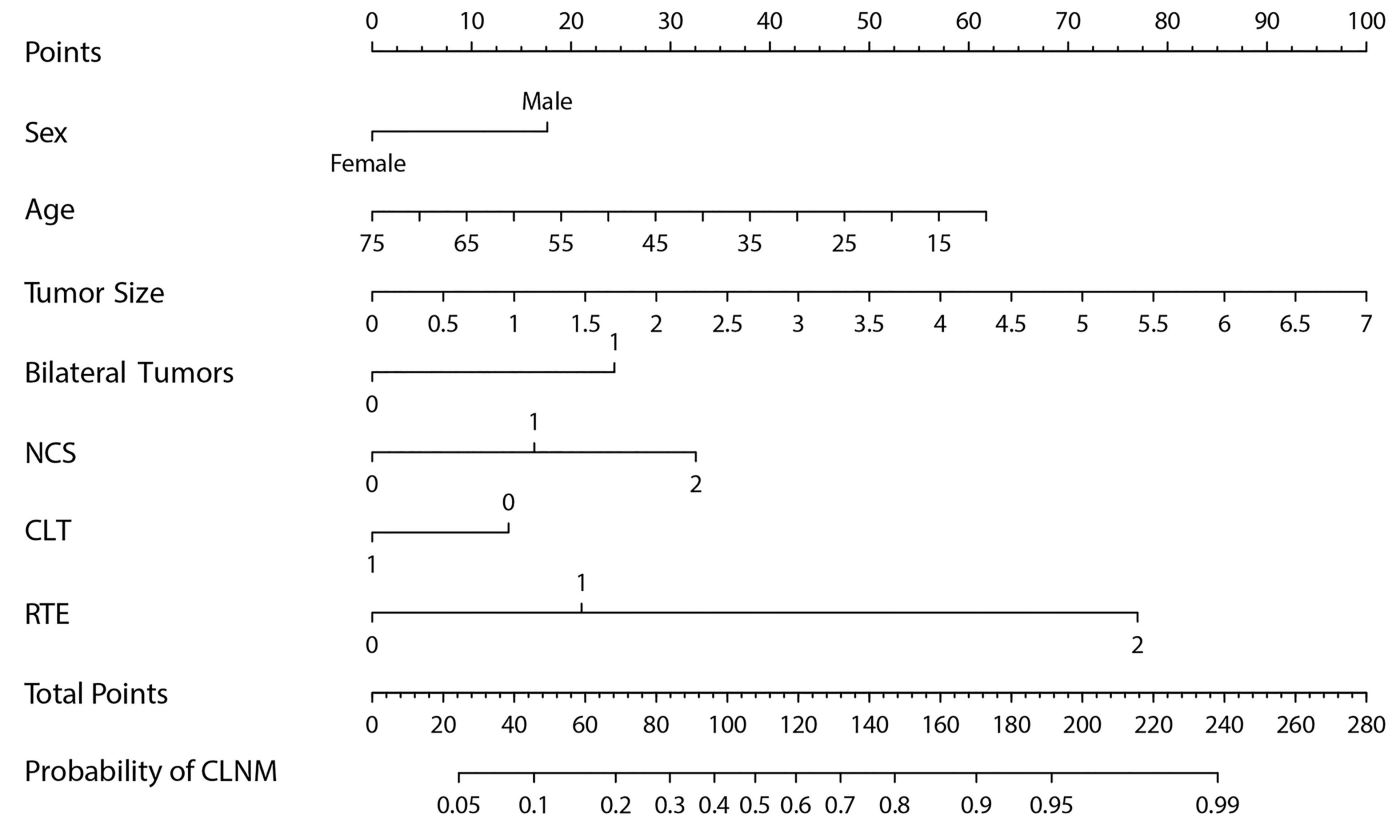
The nomogram of model 1 for predicting the risk of CLNM in cN0 PTC.

The AUC of prediction model 1 in the training dataset was 0.798, and the AUC of model 2 was 0.733 (*Z* = 5.035, *p* < 0.001, **Figure 2**); the AUC of prediction model 1 in the validation dataset was 0.792, and the AUC of model 2 was 0.715 (*Z* = 4.1895, *p* < 0.001, **Figure 3**). Comparison of the two AUCs shows that the prediction capability of model 1 was better and that RTE can substantially improve the prediction performance. Therefore, model 1 was selected as the prediction model to plot the nomogram (**Figure 4**).

### Capability of the Superior US-Based Nomogram

**Figure 5:**
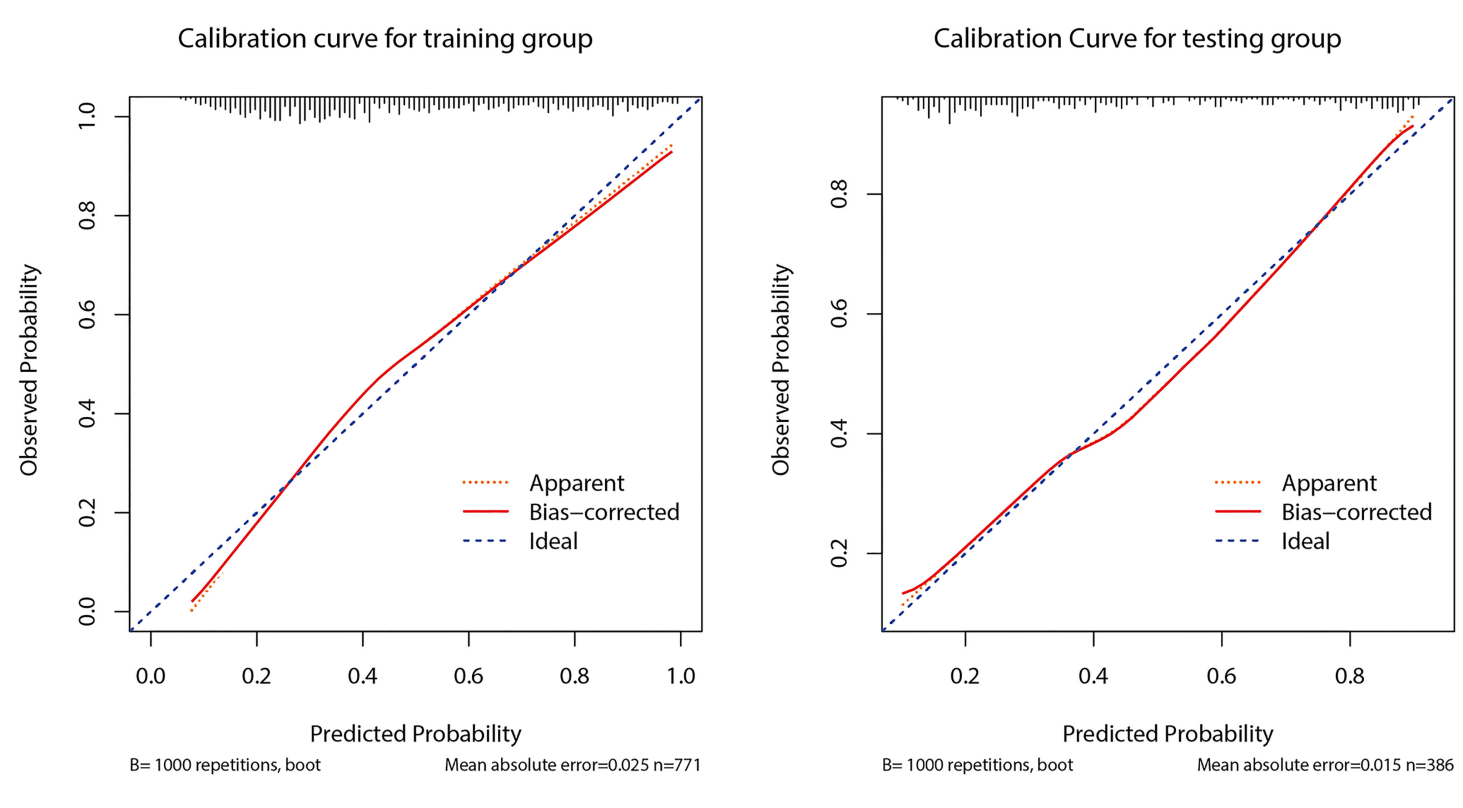
Calibration curves of the prediction model 1 in the development and validation cohorts.

**Figure 5** shows the calibration curve of prediction model 1 used to predict CLNM. The calibration curve was more consistent between predicted values and actual values in the training dataset. The Hosmer-Lemeshow goodness-of-fit test exhibited no significant difference (*p* = 0.489). The curve indicated that the predicted model almost did not deviate from the ideal model. The Hosmer-Lemeshow goodness-of-fit test also presented no significant difference in the validation cohort (*p* = 0.296). The predicted and actual values for the incidence of CLNM had a high degree of fit. When the predicted probability was between 30% and 60%, model 1 slightly overestimated the prevalence in the training set but underestimated the prevalence in the validation set.

The optimizing cutoff point of the nomo-risk was proved to be 0.407. The AUC for discriminating the incidence of CLNM in cN0 PTC patients were 0.798 (0.768–0.829) in the development group (**Figure 2**) and 0.792 (0.746–0.837) in the validation group, respectively (**Figure 3**). **Table 3** demonstrates the capability of the prime cutoff value in the nomo-risk.

**Table 3:** Performance of the prediction model for assessing the risk of CLNM.

Variable	Value (95% CI)		
	Development cohort (*n* = 771)	Validation cohort (386)	Combined cohorts (*n* = 1,157)
AUC	0.798 (0.768, 0.829)	0.792 (0.746, 0.837)	0.796 (0.771, 0.821)
Cutoff value	0.407	0.407	0.407
Sensitivity (%)	76.49 (71.83, 80.72)	77.59 (70.66, 83.55)	76.84 (73.06, 80.32)
Specificity (%)	69.58 (64.82, 74.04)	66.51 (59.72, 72.83)	68.52 (64.67, 72.18)
PPV (%)	69.88 (65.15, 74.31)	65.53 (58.61,72)	68.41 (64.56, 72.08)
NPV (%)	76.23 (71.53, 80.5)	78.33 (71.59, 84.12)	76.92 (73.16, 80.39)
Positive likelihood ratio	2.51 (2.15, 2.95)	2.32 (1.89, 2.85)	2.44 (2.15, 2.77)
Negative likelihood ratio	0.34 (0.28, 0.41)	0.34 (0.25, 0.45)	0.34 (0.29, 0.4)
Diagnosed accuracy (%)	72.89 (69.61, 76)	71.50 (66.72, 75.96)	72.43 (69.76, 74.99)

PPV, positive predictive value; NPV, negative predictive value.

### Decision Curve Analysis

**Figure 6:**
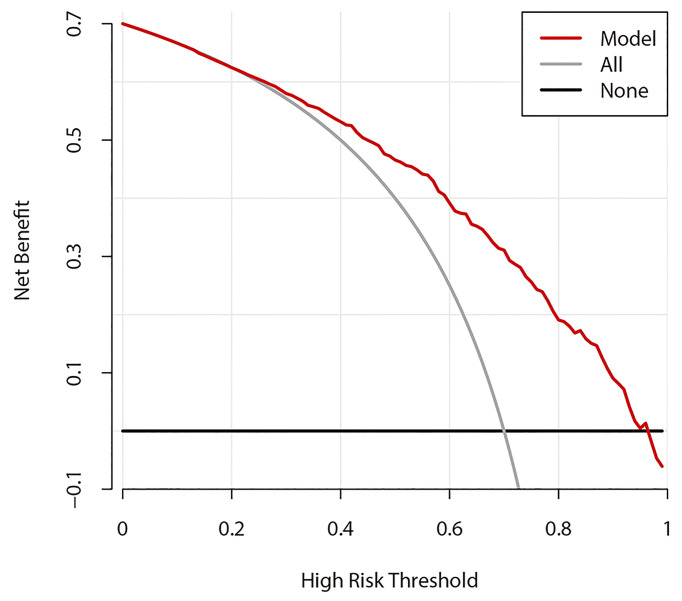
Decision curve analysis of prediction model 1.

**Figure 6** shows the DCA results of prediction model 1. The abscissa represents the threshold probability, and the value on the ordinate represents the net benefit. The prediction model is indicated by a red line. The gray line assumes that all patients had CLNM, while the black line assumes that no patients had CLNM. The calculation method for the net benefit was the true-positive rate − the false-positive rate * Pt/(1 − Pt), where Pt is the threshold probability. Based on DCA, when the threshold probability is between 22% and 95%, the prediction model can yield a good net benefit.

### US-Based Dynamic Nomogram

**Figure 7:**
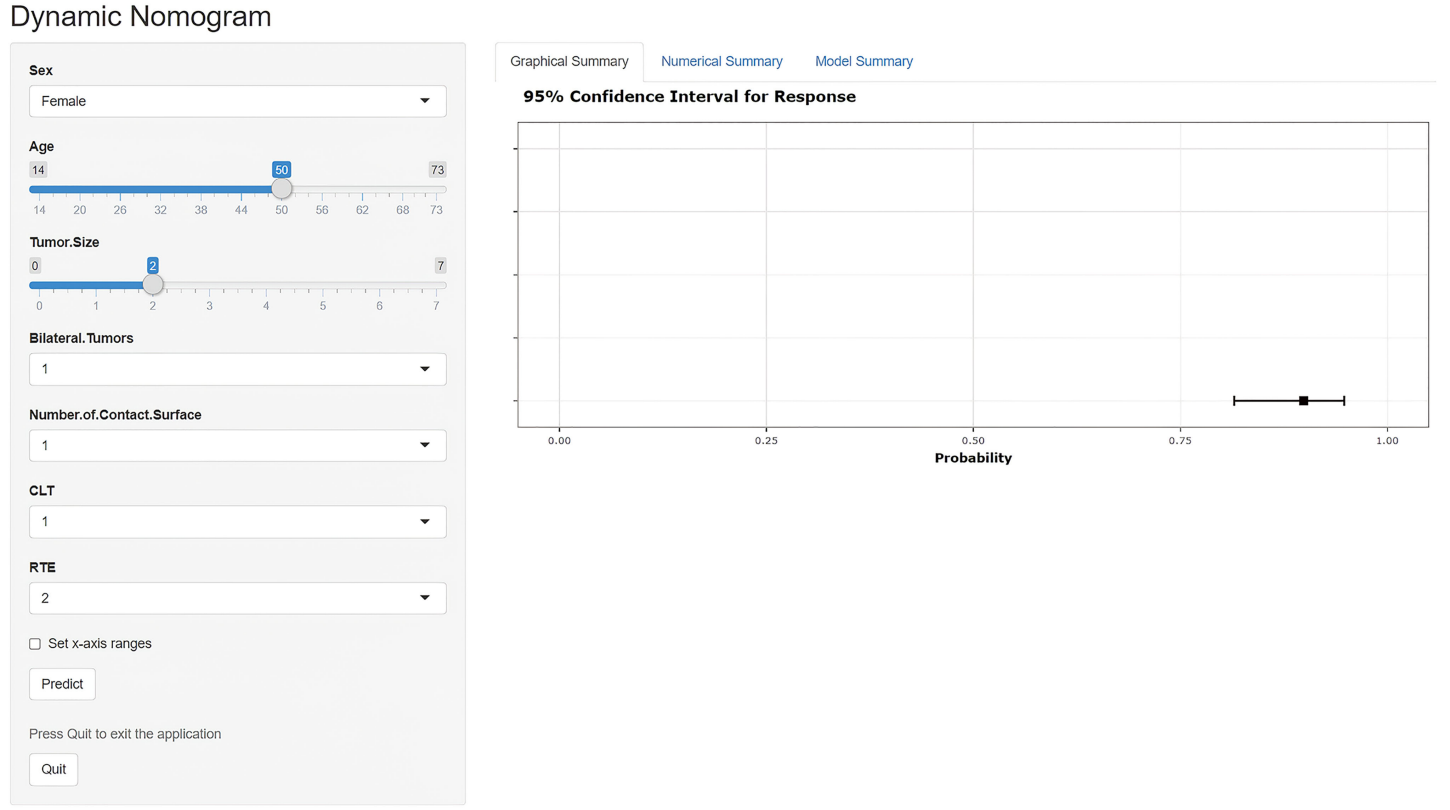
Screen shot of the dynamic US-based nomogram used to predict central lymph node metastasis in cN0 papillary thyroid carcinoma (https://predictclnminptc.shinyapps.io/DynNomapp/).

Based on the superior nomogram, a web-based dynamic probability calculator (https://predictclnminptc.shinyapps.io/DynNomapp/) was constructed to forecast the probability of CLNM in cN0 PTC patients (**Figure 7**). It is very advantageous to input the risk predictors on the web-based nomogram for real-time individualized prediction of patient’s CLNM probability. The black line, for example, represents the probability (89.9%) and 95% CI (0.815–0.948) of CLNM in the cN0 PTC patient who is female, 50 years old, tumor diameter of 2 cm, bilateral tumors, NCS = 1, CLT positive, and ES 4 (**Figure 7**).

## Discussion

PTC is known to have a high rate of CLNM, and LN metastasis is regarded as a risk factor for a poor outcome and recurrence. Therefore, LN metastasis is one of the most significant factors for clinical decision-making and patient anxiety. US is recommended as the favorite technique to appraise thyroid nodules and cervical LNs. However, the sensitivity of preoperative US for CLNM is unsatisfactory (16–19). Therefore, determining how to improve CLNM detection on US examination before surgery is particularly important. This study combined the preoperative US characteristics of PTC and clinical risk factors to establish a realistic and visual nomogram, which was converted into a web-based calculator to help clinicians and even patients with CLNM risk prediction and stratification, thereby facilitating individualized forecast of preoperative CLNM in cN0 PTC patients. Previous studies have developed nomograms to forecast the possibility of CLNM in PTC patients, which comprised postoperative pathological and clinical features but did not include preoperative US characteristics, and no web-based dynamic calculator has been developed (23, 25). As far as we know, this study is the beginner to create a web-based dynamic nomogram to predict the risk of CLNM in cN0 PTC patients.

Through multivariable logistic regression analysis, this study found that age, sex, bilateral tumors, tumor size, the NCS, CLT, and RTE were independent risk predictors of CLNM, which can all be obtained before surgery. Prediction model 1 showed high forecasting performance in the training and the validation cohorts at the same time, such as discrimination and calibration. The CLNM rate in this study was 47.0% (544/1,157), which is basically consistent with rates reported in the literature (8). The CLNM rate in males (167/288, 58%) was significantly higher than that in females (377/869, 43.4%), *p* < 0.001. The outcomes of our study showed that both younger age and male sex were independent predictors of CLNM in cN0 PTC, which is basically consistent with the findings of other studies (14, 25, 29–33). These results indicate that the risk of CLNM in young male PTC patients is increased.

Tumor size determines the T stage. In the TNM staging system of American Joint Committee on Cancer (AJCC), the T stage of a primary tumor is determined by the size of the tumor and extrathyroidal extension (ETE) (34). In this study, tumor size was independently used to predict CLNM in cN0 PTC. A larger tumor diameter corresponds to a greater chance of regional LN metastasis, which is consistent with guidelines and other studies (30, 35). Ito et al. (35) showed that tumor size was the most crucial indicator of LN recurrence and metastasis in cN0 PTC patients. The 2015 ATA guideline suggests that any clinically LN-positive patients should be treated with therapeutic CCND. When no evidence of LN metastasis is found, patients who have T3 or T4 primary thyroid tumor should be treated with prophylactic CCND. Therefore, prophylactic CCND should be considered for large-sized cN0 PTC tumor to reduce the risk of LN metastasis or recurrence. If cervical LN status is known preoperatively, the information obtained will further guide treatment measures (5).

Preoperative US evaluation of the thyroid capsule is valuable for further clinical treatment. ETE on microscope can be reliably ruled out by US to avoid possible total thyroidectomy (36). A recent study showed that US identified the status of tumors contacting thyroid capsule was significantly associated with CLNM (14). As far as we know, there are no researches that have investigated the number of thyroid tumor contacting capsule on US yet. Due to the overlap between the NCS and ETE evaluated by US, there are many literatures focusing on the ETE assessed by US. In this study, the NCS was selected as a new observation indicator, and the performance of the NCS in forecasting the CLNM hazard was evaluated. Our results showed that the NCS is an independent predictive factor of CLNM. With an increase in the NCS, the possibility of CLNM also increases.

US elastography is a promising technique that is quick and easy to accomplish and useful to recognize potentially malignant thyroid nodules and even help predict CLNM (28). Guo et al. (30) used shear wave elastography (SWE) to predict CLNM, and Young’s modulus, US features, and clinical data were integrated into a multiple regression equation for CLNM prediction, which yielded an AUC of 0.827. However, the sample size in Guo’s research was not large, and the regression equation is not convenient for clinical application. Jiang et al. (37) used a radiomics tactic to explore CLNM in PTC patients, and the multivariate regression analysis showed that the SWE radiomics score, multifocality, and US-determined LN status were independent predictive factors related to cervical LN status. Similarly, the sample size in Jiang’s study was small, and radiomics methods, which are used in only a few hospitals and are mostly in the research stage, cannot yet be applied on a large scale. A recent meta-analysis showed that RTE diagnosed better than SWE in distinguishing benign and malignant nodules (38). In terms of CLNM prediction, no literature on CLNM prediction by RTE is available. Our study shows that RTE can improve the performance of the prediction model (the AUC of model 1 in the training dataset was 0.798, while that of model 2 was 0.733, *p* < 0.001). Furthermore, the possibility of CLNM in cN0 PTC patients rose with increasing elasticity scores, and an ES of 4 had the strongest predictive performance. Tumor progression is accompanied by cell proliferation and fibrosis, which may influence the hardness and invasiveness of the tumor, including the evolution of lymph node metastasis (39).

The prevalence of PTC accompanied by CLT increased, but the correlation between CLT and CLNM in PTC patients is debatable (40, 41). A recent study concluded that CLT and CLNM were not significantly correlated with each other (40). However, some scholars hold different opinions (25, 42). One study revealed that PTC patients with CLT had a lower incidence of LN metastasis (12.2% vs. 29.9%) and showed a better prognosis in the field of mortality and recurrence (41). Our study showed that CLT on US was negatively correlated with CLNM in cN0 PTC patients. CLT may be an independent negative predictive factor of CLNM, which is consistent with the conclusions of other studies (25, 42).

In multiple studies, multifocality is considered one of the major risk factors for CLNM prediction (30, 37). Bilateral tumors are part of multifocality and are more detailed. Patients with bilateral tumors are more likely to have extrathyroidal invasion, CLNM (43), and a worse outcome (44) than patients with unilateral multifocality (45). The univariate analysis in this study showed that both multifocality and bilateral tumors were significantly correlated with CLNM. However, the bilateral multifocality was screened by multivariate logistic regression analysis for incorporation into the prediction model and achieved higher predictive performance. Because bilateral multifocality indicates that tumors are more widely distributed, this feature can predict the hazard of recurrence and mortality in PTC patients. Therefore, patients with bilateral tumors should be actively treated with an intensive follow-up (46).

This study established and validated a nomogram according to US features and clinical risk predictors for personalized prediction of the probability of CLNM in cN0 patients. The US-based nomogram yielded a satisfactory performance and permitted clinicians to forecast the possibility of CLNM development before surgery, which is in accordance with the current tendency of individualized medical management (22). Clinicians can implement prophylactic CCND for cN0 PTC patients who are forecasted to have high-risk CLNM before surgery (25).

Some limitations exist in our study. Firstly, our research is a retrospective study, and there may be selection bias. Second, retrospective studies may miss some important real-time US findings that can provide valuable diagnostic information, which in turn affects researchers’ assessments. Finally, the research data, including the validation dataset, were from a single center. Therefore, although our study preliminarily proposes that the combination of US characteristics and clinical information can predict the possibility of CLNM in cN0 PTC patients, prospective multicenter studies with larger sample sizes are still required to validate the US-based nomogram.

In summary, this study exploited a noninvasive web-based nomogram combining US characteristics and clinical risk factors. RTE could improve model prediction accuracy and the dynamic nomogram had good performance for preoperative prediction of the probability of CLNM in cN0 PTC patients.

## Data Availability Statement

The raw data supporting the conclusions of this article will be made available by the authors, without undue reservation.

## Ethics Statement

The studies involving human participants were reviewed and approved by the Research Ethics Committee of Guangdong Provincial People’s Hospital, Guangdong Academy of Medical Sciences. The ethics committee waived the requirement of written informed consent for participation.

## Author Contributions

CH, SC, and WX designed this study. WY, YW, HG, KL, and WX collected the data. CH, WY, SZ, and SC analyzed the data. All authors contributed to the article and approved the submitted version.

## Funding

This work was supported by the Guangzhou Municipal Science and Technology Planning Project (CN) (202002030235, 201804010105), Medical Scientific Research Foundation of Guangdong Province (A2019080).

## Conflict of Interest

The authors declare that the research was conducted in the absence of any commercial or financial relationships that could be construed as a potential conflict of interest.

## Publisher’s Note

All claims expressed in this article are solely those of the authors and do not necessarily represent those of their affiliated organizations, or those of the publisher, the editors and the reviewers. Any product that may be evaluated in this article, or claim that may be made by its manufacturer, is not guaranteed or endorsed by the publisher.
